# From Epidemics to Pandemics: Over a Decade of Strengthening SARInet Laboratory Surveillance and Response for Respiratory Viruses in the Americas

**DOI:** 10.1093/infdis/jiaf046

**Published:** 2025-03-10

**Authors:** Juliana Almeida Leite, Priscila S Born, Ángel Rodríguez, Paula Couto, Mauricio Cerpa, Jean-Marc Gabastou, Lionel Gresh, Leticia Franco, Mariela G Martínez, Madelaine Sugasti, Jorge H Jara, Lidia Rodondo-Bravo, Iyanna Wellington Perkins, Andrea Patricia Villalobos Rodríguez, Rosa Ramirez, Ximena Terrazas, Juliana Barbosa, Marc Rondy, Andrea S Vicari, Sylvain Aldighieri, Richard Webby, Wenqing Zhang, Dimitry Pereyaslov, Magdi Samaan, Jairo Méndez-Rico, SARInet plus laboratory network, Jairo Méndez-Rico, Juliana Almeida Leite, Priscila S Born, Andrea Vicari, Elsa Baumeister, Andrea Pontoriero, Alicia Camara, Andrea Lerman, Osvaldo Uez, Indira Martin, Draven Johnson, Songee Beckles, Kasandra Forde, Aldo Sosa, Ruby Aguillon, Roxana Loayza, Cinthia Avila, Marilda Mendonça Siqueira, Fernando Couto Motta, Paola Cristina Resende, Luana Soares Barbagelata, Terezinha Maria de Paiva, SueMin Nathaniel, Risha Singh, Rodrigo Fasce, Patricia Bustos, Sergio Yebrail Gomez Rangel, Paula Estefania Rodriguez Romero, Hebleen Brenes, Jorge Sequeira-Soto, María Guadalupe Guzmán, Elias Guilarte Garcia, Catherina Jemmott, Eric Carbon, Lucía de la Cruz, Nurys de Castro, Alfredo Bruno, Doménica de Mora Coloma, Dalia Xochitl Sandoval López, Denis Gerson Jovel Alvarado, Selene González, Christa Leal, Joyce Whyte-Chin, Mustapha Abdul-Kadir, Jacques Boncy, Ito Journel, Mitzi Castro Paz, Dulce María Durón, Michelle Brown, Suwanie Lewis, Irma Lopez Martinez, Gisela Barrera-Badillo, Brechla Moreno, Danilo Franco, Cynthia Vazquez, María José Ortega, Johanna Balbuena-Torres, Maribel Huaringa, Candace Gumbs, Andrea Williams, Joseph N France, Wayne Felicien, Vernel Feloion, Phyllis S Pinas, Merissa A Garraway-Miller, Arlene Siebs, Hector Chiparelli, Natalia Goñi, Lieska Rodriguez, Pierina D’Angelo

**Affiliations:** Infectious Hazard Management Unit, Health Emergencies Department, Pan American Health Organization, Washington, DC; Infectious Hazard Management Unit, Health Emergencies Department, Pan American Health Organization, Washington, DC; Infectious Hazard Management Unit, Health Emergencies Department, Pan American Health Organization, Washington, DC; Infectious Hazard Management Unit, Health Emergencies Department, Pan American Health Organization, Washington, DC; Health Emergencies, Pan American Health Organization, Bogota, Colombia; Infectious Hazard Management Unit, Health Emergencies Department, Pan American Health Organization, Washington, DC; Infectious Hazard Management Unit, Health Emergencies Department, Pan American Health Organization, Washington, DC; Infectious Hazard Management Unit, Health Emergencies Department, Pan American Health Organization, Washington, DC; Infectious Hazard Management Unit, Health Emergencies Department, Pan American Health Organization, Washington, DC; Infectious Hazard Management Unit, Health Emergencies Department, Pan American Health Organization, Washington, DC; Comprehensive Immunization Program, Pan American Health Organization, Washington, DC; Infectious Hazard Management Unit, Health Emergencies Department, Pan American Health Organization, Washington, DC; Infectious Hazard Management Unit, Health Emergencies Department, Pan American Health Organization, Washington, DC; Infectious Hazard Management Unit, Health Emergencies Department, Pan American Health Organization, Washington, DC; Infectious Hazard Management Unit, Health Emergencies Department, Pan American Health Organization, Washington, DC; Infectious Hazard Management Unit, Health Emergencies Department, Pan American Health Organization, Washington, DC; Health Emergencies, Pan American Health Organization, Bogota, Colombia; Infectious Hazard Management Unit, Health Emergencies Department, Pan American Health Organization, Washington, DC; Infectious Hazard Management Unit, Health Emergencies Department, Pan American Health Organization, Washington, DC; Department of Prevention, Control and Elimination of Communicable Diseases, Pan American Health Organization, Washington, DC; Department of Infectious Diseases, St Jude Children's Research Hospital, Memphis, Tennessee; WHO Health Emergencies Programme, World Health Organization Headquarters, Geneva, Switzerland; WHO Health Emergencies Programme, World Health Organization Headquarters, Geneva, Switzerland; WHO Health Emergencies Programme, World Health Organization Headquarters, Geneva, Switzerland; Infectious Hazard Management Unit, Health Emergencies Department, Pan American Health Organization, Washington, DC

**Keywords:** influenza, International Health Regulations, preparedness and response, Laboratory, SARS-CoV-2

Many efforts have been made to predict and control emerging infectious disease threats to human health. However, it is still a challenge to predict the next causal agent. In the past decades, numerous emerging and/or reemerging respiratory viruses have affected humans: severe acute respiratory syndrome (SARS; 2003), influenza A(H1N1) (2009), Middle East respiratory syndrome (2012), and SARS-CoV-2 (2019) [1]. Several respiratory viruses, including influenza viruses, have epidemic and/or pandemic potential [2, 3]. For this reason, the World Health Organization (WHO) Global Influenza Surveillance and Response System (GISRS), established in 1952, has recently been expanded to e-GISRS to monitor and respond to any epidemic and pandemic threat posed not only by influenza viruses but also by other respiratory viruses [4, 5].

This perspective discusses improvements in laboratory capacity building and genomic surveillance for respiratory viruses in the Americas. It emphasizes the crucial role of the Pan American Health Organization (PAHO) and its networks in strengthening public health responses and building regional resilience against emerging and/or reemerging respiratory threats.

## A HISTORICAL PERSPECTIVE: SARINET’S CONTRIBUTIONS TO RESPIRATORY VIRUS SURVEILLANCE

**Figure 1.**
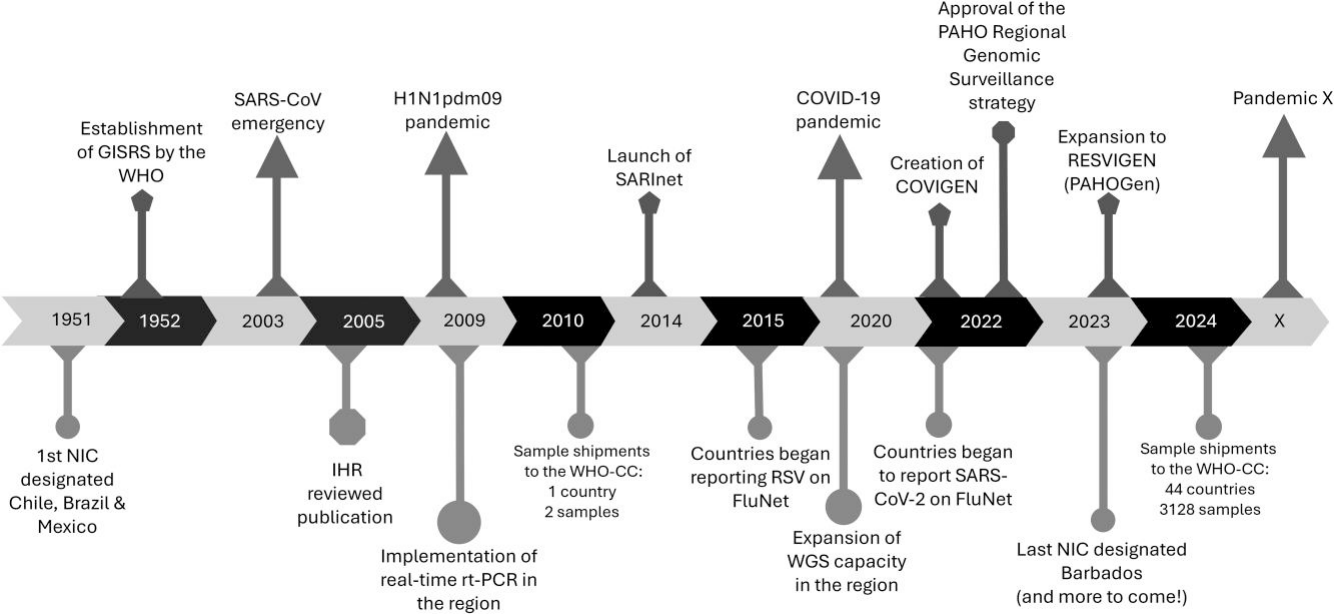
Hallmark events on laboratory surveillance and response for respiratory viruses in the Americas. Abbreviations: COVIGEN, COVID-19 Genomic Surveillance Regional Network; GISRS, Global Influenza Surveillance and Response System; IHR, international health regulations; NIC, National Influenza Centre; PAHOGen, Pan American Health Organization Genomic Surveillance Regional Networks; RESVIGEN, Respiratory Virus Genomic Surveillance Regional Network; RT-PCR, reverse transcription polymerase chain reaction; RSV, respiratory syncytial virus; SARInet, Severe Acute Respiratory Infections network; WGS, whole genome sequencing; WHO-CC, World Health Organization Collaborating Centre.

The first National Influenza Centres (NICs) in the Americas were established in 1951, with laboratories from Brazil, Chile, and Mexico invited to be part of global influenza surveillance prior to the creation of GISRS. Over the following decades, these NICs expanded their capacities and served as models for the establishment of additional NICs across the region, supporting the foundation of robust influenza surveillance systems. By 2009, NICs were operating in 22 countries, enabling enhanced detection and response capabilities. The first pandemic of this century, caused by the influenza virus A(H1N1pdm) in 2009, significantly affected the Americas region, with all 35 countries confirming cases. By 2 October 2009, there were >146 000 laboratory-confirmed cases in all 35 countries and 3292 confirmed deaths in 25 countries in the region [6]. After this pandemic, countries of the Americas recognized the need to develop and implement surveillance of severe acute respiratory infections in sentinel hospitals in the region [7]. To address this need, the Severe Acute Respiratory Infections Network (SARInet) was established by the PAHO and its member states in 2014 (Figure 1). SARInet also aimed to enhance laboratory surveillance for respiratory viruses in the region. Since then, the extensive network of SARInet laboratories serves as the backbone for respiratory virus surveillance [8]. The network is supported by the PAHO, WHO, and WHO Collaborating Centres in the Americas region at the US Centers for Disease Control and Prevention (CDC) and at the St Jude Children's Research Hospital.

**Figure 2.**
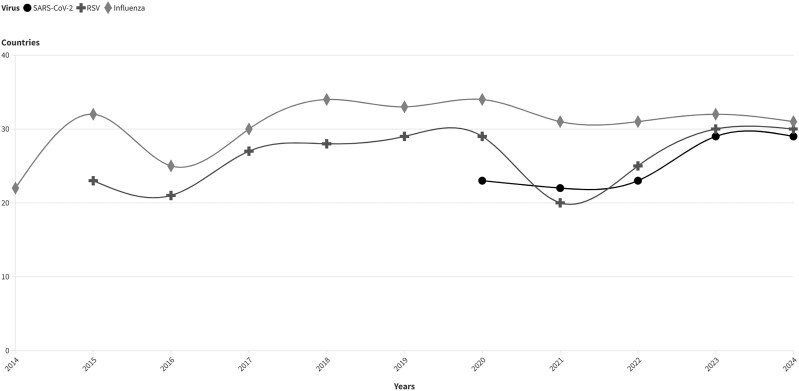
Number of countries reporting through FluNet, by respiratory virus and year. Source: FluNet. Abbreviations: RSV, respiratory syncytial virus.

When SARInet was launched, 22 countries were reporting influenza virus data through FluNet, a global tool for influenza virologic surveillance initiated by the WHO in 1997 [9]. As of August 2024, the SARInet laboratory network includes 31 NICs and 13 national reference laboratories, allowing 30 countries to regularly report data for influenza and other respiratory viruses via FluNet (Figure 2). This reporting capability has been essential in enhancing regional surveillance; however, barriers persist for some countries. Challenges such as a lack of trained personnel, sample transportation logistical issues, and communication gaps between laboratory and epidemiology teams continue to hinder consistent data submission and disrupt the flow of timely and accurate data. The PAHO has implemented targeted initiatives to address these challenges (eg, training), enhanced logistics support to the countries (eg, dry ice machines), and improved communication through meetings with different sectors involved. An integrated information system, PAHOFlu, has been developed to enhance real-time monitoring, analysis, and sharing of data on influenza and other respiratory viruses among ministries of health, sentinel sites, and laboratories. These efforts ensure that more countries can participate fully in FluNet reporting, enhancing the region's preparedness and response capabilities, as demonstrated during the COVID-19 pandemic. SARInet laboratories are part of the WHO e-GISRS, and when the COVID-19 pandemic began in 2020, the existing capability to perform virologic surveillance in the region was crucial for the response. In 2022, SARInet expanded its surveillance to include other respiratory viruses with epidemic and pandemic potential, with a change of name to SARInet plus. It now integrates surveillance for influenza, SARS-CoV-2, and other respiratory viruses [10].

SARInet laboratories conduct high-quality virologic surveillance for respiratory viruses, with virus detection and antigenic and genetic characterization. They share crucial virologic data globally through FluNet and send virus samples to the WHO Collaborating Centres for generating information for both influenza vaccine compositions: seasonal and zoonotic. Additionally, these laboratories contribute to the characterization of other respiratory virus strains, promote collaborations among laboratories inside the Americas region, and participate in global efforts to the overall surveillance and response framework in the region, particularly for influenza, SARS-CoV-2, and respiratory syncytial virus [3, 4]. Their participation in the WHO Influenza and SARS-CoV-2 External Quality Assurance Program ensures the accuracy and reliability of laboratory testing through regular assessments and proficiency testing [11].

## FROM PREPAREDNESS TO ACTION: SARINET’S IMPACT ON PANDEMIC RESPONSE

Pandemic preparedness for respiratory viruses in the region began with GISRS, emphasizing early detection of novel influenza strains. The 2003 SARS-CoV outbreak highlighted the need to strengthen epidemiologic and laboratory capacities to rapidly respond to emerging infections [12]. In response, the international health regulations were revised in 2005 to coordinate global efforts, enhancing early detection, reporting, and response to infectious diseases and other public health emergencies. SARInet laboratories have played a critical role in responding to epidemics and pandemics throughout the Americas for more than a decade. During the 2009 influenza pandemic, laboratories transitioned from immunofluorescence assays to molecular diagnostics, adopting high-quality standardized real-time reverse transcription polymerase chain reaction (RT-PCR) assays. This shift enabled not only the rapid and accurate detection of influenza A(H1N1)pdm09 virus but also the detection of influenza A and B viruses and the subtyping of seasonal and zoonotic influenza A viruses, significantly enhancing regional response capabilities [13]. In 2013, the laboratories in the Americas demonstrated readiness during the Middle East respiratory syndrome outbreak by promptly implementing molecular techniques, effectively covering the diagnosis of the novel coronavirus within the region [14]. During the COVID-19 pandemic, through a PAHO-coordinated strategy and collaborative work, >29 countries expanded their diagnostic capabilities for SARS-CoV-2 detection before the WHO declaration of the COVID-19 pandemic [15]. Furthermore, by the end of 2020, SARInet laboratories could implement multiplex diagnostic assays from the CDC, enabling simultaneous detection of multiple influenza and SARS-CoV-2 viruses. At the same time, the COVID-19 response allowed for the expansion of molecular testing capacity to many countries and territories in the Caribbean, which had no such capacity before.

Until 2019, the Caribbean subregion had NICs in Cuba, Haiti, Dominican Republic, Jamaica, French Guyana, Suriname, and CARPHA (Caribbean Public Health Agency) with molecular capability to test influenza viruses including H5 and H7. During the pandemic response, SARS-CoV-2 molecular capacity was rapidly established in all countries in the Caribbean subregion, and notably, open-platform molecular laboratories were established for the very first time in 5 countries (Antigua and Barbuda, Grenada, Saint Kitts and Nevis, Saint Lucia, and Saint Vincent and the Grenadines). This capacity was then leveraged from 2022 to 2024 to strengthen the subregion by implementing influenza laboratory surveillance using protocols of WHO Collaborating Centre at US-CDC in these 5 countries as well as Guyana.

Today, respiratory virus samples are routinely tested in parallel for influenza and SARS-CoV-2 by reagents for the integrated surveillance made available through the WHO Collaborating Centres at the CDC International Reagent Resource. Having sustainable distribution of diagnostic tests is crucial to ensure the continued diagnosis of respiratory virus in the region.

Moreover, classical virologic methods, such as antigenic characterization and phenotypic antiviral testing, are predominantly performed at WHO Collaborating Centres, reflecting the financial, technical, and infrastructure challenges associated with these techniques and to ensure high-quality analyses. This has led to a regional focus on molecular diagnostics, such as RT-PCR and whole genome sequencing, which are more feasible for routine laboratory operations and scalable to meet demand. These methods are supported by SARInet plus through training and resource provision, which has significantly enhanced regional diagnostic capacity and efficiency. As the need for SARS-CoV-2 testing decreased, these newly established molecular platforms were leveraged for influenza—including influenza A(H5), which was recently introduced in the region—and other pathogens of public health importance testing.

Genomic surveillance, previously used in the network for the characterization of influenza viruses, proved to be pivotal during the COVID-19 pandemic. Significant advancements were made in upgrading and integrating advanced sequencing technologies during that time [15, 16]. Early into the pandemic response, in March 2020, the PAHO established the COVID-19 Genomic Surveillance Regional Network (COVIGEN) [15]. This network supported >15 countries with existing sequencing capacity to rapidly implement SARS-CoV-2 whole genome sequencing by providing protocols, training, and reagents [15]. Moreover, the network allowed countries without sequencing capacity to ship samples to 1 of the 8 COVIGEN reference sequencing laboratories. As a result, >43% of all SARS-CoV-2 genomes from Latin America and the Caribbean were generated in laboratories participating in the COVIGEN network, showing the importance of international collaboration networking (data analyzed from the GISAID database, https://gisaid.org/). This genomic surveillance proved instrumental in tracking viral evolution, identifying variants of concern, and guiding public health interventions [15, 17]. In 2023, COVIGEN expanded into the Respiratory Virus Genomic Surveillance Regional Network, fostering national and regional laboratory collaborations to encompass influenza, SARS-CoV-2, respiratory syncytial virus, and other respiratory viruses to enhance epidemiologic understanding and optimize control strategies [18]. All these efforts are part of the “Strategy on Regional Genomic Surveillance for Epidemic and Pandemic Preparedness and Response,” endorsed in 2022 by the 30th Pan American Sanitary Conference, and underscore the importance for the region to implement and expand these key tools for early detection and monitoring of viruses that pose significant health threats [19].

Moreover, SARInet plus supports training initiatives to fortify readiness and enhance laboratory proficiency in molecular diagnostics and genomic sequencing. Workshops, hands-on training, and online courses have standardized protocols and equipped laboratories with cutting-edge technologies, ensuring swift and accurate responses to emerging threats [8].

The collective efforts at the regional level have significantly expanded laboratory capacity for molecular testing and characterization. In terms of quality, the number of NICs and national reference laboratories participating in the WHO external quality assessment program for influenza virus detection by RT-PCR has increased from 32 in 2016 to 45 in 2023. This expansion follows the increase in the number of NICs and National Public Health Laboratories performing RT-PCR (Table 1) and is remarkable in the Caribbean subregion, where several countries implemented molecular testing for the first time as a response to the pandemic and are now using the capacity to test for influenza and other respiratory viruses, and other endemic and emerging pathogens as monkeypox virus and arboviruses.

**Table 1.** Respiratory Virus Capabilities of the NICs and National Reference Laboratories of the PAHO Region in 2014 and 2024

	NIC Status		EQAP Participation		Molecular Diagnostic^a^		Sequencing Capacity^b^		FluNet Reporting^c^	
Region: Country	2014	2024	2014	2024	2014	2024	2014	2024	2014	2024
**North America**										
Canada	**Yes**	**Yes**	**Yes**	**Yes**	**Yes**	**Yes**	**Yes**	**Yes**	**Yes**	**Yes**
Mexico	**Yes**	**Yes**	**Yes**	**Yes**	**Yes**	**Yes**	**Yes**	**Yes**	**Yes**	**Yes**
United States	**Yes**	**Yes**	**Yes**	**Yes**	**Yes**	**Yes**	**Yes**	**Yes**	**Yes**	**Yes**
**Caribbean**										
Bahamas	No	No	No	**Yes**	No	**Yes**	No	No	No	No
Barbados	No	**Yes**	**Yes**	**Yes**	No	**Yes**	No	**Yes**	No	**Yes**
Belize	No	No	No	**Yes**	**Yes**	**Yes**	No	No	No	**Yes**
Cayman Islands	No	No	No	**Yes**	No	**Yes**	No	No	No	**Yes**
Cuba	**Yes**	**Yes**	**Yes**	**Yes**	**Yes**	**Yes**	No	**Yes**	**Yes**	**Yes**
Dominica	No	No	No	**Yes**	No	**Yes**	No	No	No	No
Dominican Republic	No	**Yes**	**Yes**	**Yes**	**Yes**	**Yes**	No	**Yes**	**Yes**	**Yes**
Grenada	No	**Yes**	No	**Yes**	No	**Yes**	No	No	No	No
Guyana	No	No	No	**Yes**	No	**Yes**	No	**Yes**	No	**Yes**
Haiti	No	**Yes**	No	**Yes**	No	**Yes**	No	**Yes**	No	**Yes**
Jamaica	**Yes**	**Yes**	**Yes**	**Yes**	**Yes**	**Yes**	No	**Yes**	**Yes**	**Yes**
Saint Lucia	No	No	No	**Yes**	No	**Yes**	No	No	No	**Yes**
St Vincent and the Grenadines	No	No	No	**Yes**	No	**Yes**	No	No	No	**Yes**
Suriname	No	**Yes**	**Yes**	**Yes**	No	**Yes**	No	No	No	**Yes**
Trinidad and Tobago (CARPHA)	**Yes**	**Yes**	**Yes**	**Yes**	**Yes**	**Yes**	No	No	No	**Yes**
**Central America**										
Costa Rica	**Yes**	**Yes**	**Yes**	**Yes**	**Yes**	**Yes**	**Yes**	**Yes**	**Yes**	**Yes**
El Salvador	**Yes**	**Yes**	No	**Yes**	**Yes**	**Yes**	No	**Yes**	**Yes**	**Yes**
Guatemala	**Yes**	**Yes**	**Yes**	**Yes**	**Yes**	**Yes**	No	**Yes**	**Yes**	**Yes**
Honduras	**Yes**	**Yes**	No	**Yes**	**Yes**	**Yes**	No	**Yes**	**Yes**	**Yes**
Nicaragua	**Yes**	**Yes**	**Yes**	**Yes**	**Yes**	**Yes**	No	**Yes**	**Yes**	**Yes**
Panama	**Yes**	**Yes**	No	**Yes**	**Yes**	**Yes**	**Yes**	**Yes**	**Yes**	**Yes**
**Andean**										
Bolivia	No	**Yes**	**Yes**	**Yes**	**Yes**	**Yes**	No	**Yes**	**Yes**	**Yes**
Colombia	**Yes**	**Yes**	**Yes**	**Yes**	**Yes**	**Yes**	No	**Yes**	**Yes**	**Yes**
Ecuador	**Yes**	**Yes**	**Yes**	**Yes**	**Yes**	**Yes**	No	**Yes**	**Yes**	**Yes**
Peru	**Yes**	**Yes**	**Yes**	**Yes**	**Yes**	**Yes**	No	**Yes**	**Yes**	**Yes**
Venezuela	**Yes**	**Yes**	**Yes**	**Yes**	**Yes**	**Yes**	No	**Yes**	**Yes**	**Yes**
**Brazil and Southern Cone**										
Argentina	**Yes**	**Yes**	**Yes**	**Yes**	**Yes**	**Yes**	**Yes**	**Yes**	**Yes**	**Yes**
Brazil	**Yes**	**Yes**	**Yes**	**Yes**	**Yes**	**Yes**	**Yes**	**Yes**	**Yes**	**Yes**
Chile	**Yes**	**Yes**	**Yes**	**Yes**	**Yes**	**Yes**	**Yes**	**Yes**	**Yes**	**Yes**
Paraguay	**Yes**	**Yes**	**Yes**	**Yes**	**Yes**	**Yes**	No	**Yes**	**Yes**	**Yes**
Uruguay	**Yes**	**Yes**	**Yes**	**Yes**	**Yes**	**Yes**	**Yes**	**Yes**	**Yes**	**Yes**

Source: FluNet, Respiratory Virus Genomic Surveillance Regional Network/PAHO Genomic Surveillance Regional Networks, and Global Influenza Surveillance and Response System/WHO.

Abbreviations: CARPHA, Caribbean Public Health Agency; EQAP, External Quality Assurance Programme (Global Influenza Programme/WHO); NIC, National Influenza Centre; PAHO, Pan American Health Organization; WHO, World Health Organization. Bold values indicate key achievements in place.

^a^Molecular detection for at least influenza and SARS-CoV-2.

^b^Sequencing capacity for influenza and/or SARS-CoV-2.

^c^Weekly virologic data reporting to WHO FluNet platform.

## SHAPING THE FUTURE: COLLABORATIVE EFFORTS FOR A RESILIENT LABORATORY NETWORK

### Lessons Learned

SARInet plus laboratories have gained valuable insights into enhancing laboratory surveillance through their participation in e-GISRS. One key lesson is the importance of adopting advanced diagnostic technologies, such as real-time RT-PCR and whole genome sequencing, to improve the detection and characterization of respiratory viruses [8]. This includes adherence to international health regulations. Best practices identified through SARInet plus activities encompass the importance of data sharing, collaborative research, and implementing protocols to update diagnostics. The network's experience also highlights the significance of robust training programs to ensure standardized and efficient diagnostic practices across laboratories. These practices need to follow good laboratory practices and biosafety and biosecurity recommendations, including the WHO Global Laboratory Leadership Program, which plays a crucial role in enhancing laboratory capabilities and leadership.

### Main Challenges

Challenges to build a resilient laboratory network include resource limitations, such as insufficient human resources, variability in laboratory infrastructure, and disparities in information systems, as well as the need to improve the linking of laboratory and epidemiologic data for enhanced surveillance. Continuous training, such as bioinformatic workshops and training sessions, is crucial to keeping pace with evolving technologies. Additionally, fostering effective communication channels among laboratories has proven essential for effective response coordination during outbreaks.

While the Nagoya Protocol ensures equitable benefit sharing, it has sometimes delayed virus and sample exchange and the sharing of genetic resources, affecting research, vaccine development, and global public health initiatives. SARInet plus has been able to mitigate this by promoting One Health collaborations between animal and human health laboratories to address zoonotic threats such as avian influenza A(H5).

As expected, the critical shortages of auxiliary plastic materials as well as reagent kits which are essential for testing; along with restrictions and disruptions in sample shipping logistics also affected laboratory diagnostics across countries during the COVID-19 pandemic. The PAHO addressed these challenges by coordinating procurement and distribution of critical supplies and optimizing sample shipping logistics.

Virologic surveillance in the Americas is an essential pillar of sentinel surveillance for respiratory viruses, aligning with international guidelines for monitoring virologic and epidemiologic trends. This system operates across outpatient and hospital settings, leveraging case definitions for influenza-like illness (ILI) and severe acute respiratory infection to capture comprehensive information of respiratory virus activity. The integration of syndromic and laboratory data has provided critical insights into the burden and severity of respiratory viruses in the region. Importantly, ILI surveillance has demonstrated its value in detecting the onset of circulation seasons, often identifying mild cases before severe hospitalizations occur. Currently, 17 countries in the region report weekly ILI case data with viral confirmation. Despite the success, a significant challenge remains in increasing the number of countries implementing ILI surveillance alongside severe acute respiratory infection surveillance.

## FUTURE DIRECTIONS AND RECOMMENDATIONS

Future directions for SARInet plus laboratory surveillance in the Americas region include expanding genomic surveillance capabilities and integrating advanced bioinformatics tools to enhance data analysis. Opportunities for further collaboration with global health partners, such as e-GISRS, can facilitate knowledge exchange and resource sharing, strengthening the global surveillance network.

To strengthen laboratory capabilities and response to respiratory infections, it is recommended that more investment be made, sustainable funding for laboratory activities be ensured, and continuous professional development for laboratory personnel be prioritized. Implementing standardized protocols and fostering a culture of collaboration and innovation are also crucial steps. Furthermore, responses to previous pandemics and epidemics emphasize the importance of collaboration across sectors through a One Health approach.

Reiterating the importance of ongoing efforts to strengthen the virologic surveillance of respiratory viruses as part of countries' preparedness and readiness for responding to future epidemics and pandemics, the experiences of SARInet plus underscore the need for a well-coordinated global approach to respiratory infection surveillance. Continued collaboration with international health partners, such as the WHO Collaborating Centres and the WHO Global Influenza Programme, which includes GISRS, and with initiatives such as RESVIGEN and the PAHO Genomic Surveillance Regional Network will be vital in addressing future public health challenges. These partnerships enhance diagnostic capacity, strengthen genomic surveillance, and build resilient health systems, safeguarding communities from respiratory threats.

## Notes

***Acknowledgments.*** We are grateful for all the work of the National Influenza Centres and National Public Health Laboratories as part of SARInet plus for contributing to global respiratory virus surveillance through GISRS/WHO. We thank GISAID and all the submitters of the database, as well as to the reference sequencing laboratories of the Respiratory Virus Genomic Surveillance Regional Network for all their contributions on respiratory virus genomic surveillance. We are grateful for the support of personnel at PAHO headquarters and PAHO/WHO country offices for the technical cooperation provided in the region to all respiratory virus pandemics. We are thankful for all collaboration and support from the WHO Collaborating Centres at the CDC and St Jude Children's Research Hospital to the network. We are grateful to all the donors to PAHO, including the CDC, US Department of Health and Human Services, Wellcome Trust, and WHO.

***Author contributions.*** The authors confirm their contributions to the article as follows. Substantial contributions to the conception or design of the work: P. S. B., J. M.-R., J. A. L. Data collection: P. S. B., M. G. M., L. G. SARInet laboratory network: J. M.-R., J. A. L. Analysis and interpretation of results: P. S. B., L. R. B., J. A. L. Drafting the work: P. S. B., J. A. L., J. M.-R., M. G. M., L. G. All authors critically reviewed the results and manuscript for important intellectual content.

***SARInet laboratory network.***  *PAHO:* Jairo Méndez-Rico, Juliana Almeida Leite, Priscila S. Born, Andrea Vicari. *Antigua and Barbuda:* Sir Lester Bird Medical Center. *Argentina:* Elsa Baumeister, Andrea Pontoriero–Administración Nacional de Laboratorios e Institutos de Salud “Dr Carlos G. Malbrán”; Jorge Camara, Alicia Camara–Instituto de Virología; Facultad de Ciencias Médicas, UNC Córdoba; Andrea Lerman, Osvaldo Uez–Instituto Nacional de Epidemiología. *Bahamas:* Indira Martin, Draven Johnson–Ministry of Health Reference Laboratory. *Barbados:* Songee Beckles, Kasandra Forde–Best-Dos Santos Public Health Laboratory. *Belize:* Aldo Sosa, Ruby Aguillon–Central Medical Laboratory. *Bolivia:* Roxana Loayza, Cinthia Avila–Centro Nacional de Enfermedades Tropicales. *Brazil:* Marilda Mendonça Siqueira, Fernando Couto Motta, Paola Cristina Resende–Fundação Oswaldo Cruz; Mirleide Cordeiro dos Santos, Luana Soares Barbagelata–Instituto Evandro Chagas; Katia Corrêa de Oliveira Santos, Terezinha Maria de Paiva–Instituto Adolfo Lutz. *Canada:* National Microbiology Laboratory. *CARPHA:* SueMin Nathaniel, Risha Singh–Caribbean Public Health Agency. *Chile:* Rodrigo Fasce, Patricia Bustos–Instituto de Salud Pública de Chile. *Colombia:* Sergio Yebrail Gomez Rangel, Paula Estefania Rodriguez Romero–Instituto Nacional de Salud. *Costa Rica:* Hebleen Brenes, Jorge Sequeira-Soto–Instituto Costarricense de Investigación y Enseñanza en Nutrición y Salud. *Cuba:* María Guadalupe Guzmán, Elias Guilarte Garcia–Instituto de Medicina Tropical Pedro Kourí. *Dominica:* Catherina Jemmott, Eric Carbon–National Medical Laboratory/Dominica China Friendship Hospital. *Dominican Republic:* Lucía de la Cruz, Nurys de Castro–Laboratorio Nacional de Salud Pública Dr Defilló. *Ecuador:* Alfredo Bruno, Doménica de Mora Coloma–Instituto Nacional de Investigación en Salud Pública. *El Salvador:* Dalia Xochitl Sandoval López, Denis Gerson Jovel Alvarado–Laboratorio Nacional de Salud Publica “Dr Max Bloch.” *Grenada:* General Hospital Laboratory. *Guatemala:* Selene González, Christa Leal–Laboratorio Nacional de Salud. *Guyana:* Joyce Whyte-Chin, Mustapha Abdul-Kadir–National Public Health Reference Laboratory. *Haiti:* Jacques Boncy, Ito Journel–Laboratoire National de Santé Publique. *Honduras:* Mitzi Castro Paz, Dulce María Durón–Laboratorio Nacional de Vigilancia de la Salud. *Jamaica:* Michelle Brown, Suwanie Lewis–Virology Laboratory, University of the West Indies. *Mexico:* Irma Lopez Martinez, Gisela Barrera-Badillo–Instituto de Diagnóstico y Referencia Epidemiológicos. *Panama:* Brechla Moreno, Danilo Franco–Instituto Conmemorativo Gorgas de Estudios de la Salud. *Paraguay:* Cynthia Vazquez, María José Ortega–Laboratorio Central de Salud Pública. *Peru:* Johanna Balbuena-Torres, Maribel Huaringa–Instituto Nacional de Salud. *Saint Kitts and Nevis:* Candace Gumbs, Andrea Williams–Pathology Laboratory, Joseph N. France General Hospital. *Saint Lucia:* Wayne Felicien, Vernel Feloion–Ezra Long Laboratory. *Saint Vincent and the Grenadines:* Molecular Laboratory Unit. *Suriname:* Phyllis S. Pinas–Central Laboratory of the Bureau of Public Health. *Trinidad and Tobago:* Trinidad Public Health Laboratory. *Turks and Caicos Islands:* Merissa A. Garraway-Miller, Arlene Siebs–National Public Health Laboratory. *Uruguay:* Hector Chiparelli, Natalia Goñi–Departamento de Laboratorio de Salud Pública. *Venezuela:* Lieska Rodriguez, Pierina D’Angelo–Instituto Nacional de Higiene Rafael Rangel, Caracas.

***Financial support***. No financial support was received for this work.

***Supplement sponsorship.*** This article appears as part of the supplement “Advancing Respiratory Virus Surveillance and Preparedness in the Americas: A Decade of SARInet's Impact,” sponsored by the Pan American Health Organization.

***Potential conflicts of interest.*** All authors have submitted an ICMJE form.
